# Constraints and Affordances of Online Engagement With Scientific Information—A Literature Review

**DOI:** 10.3389/fpsyg.2020.572744

**Published:** 2020-12-08

**Authors:** Friederike Hendriks, Elisabeth Mayweg-Paus, Mark Felton, Kalypso Iordanou, Regina Jucks, Maria Zimmermann

**Affiliations:** ^1^Institute for Psychology in Education and Instruction, Department of Psychology and Sport Studies, University of Münster, Münster, Germany; ^2^Institute of Educational Studies, Faculty of Humanities and Social Sciences, Humboldt University of Berlin, Einstein Center Digital Future, Berlin, Germany; ^3^Department of Teacher Education, Lurie College of Education, San Jose State University, San Jose, CA, United States; ^4^School of Sciences, University of Central Lancashire, Larnaka, Cyprus

**Keywords:** epistemic cognition, argumentation, scientific literacy, digital literacy, multiple documents literacy, online engagement with scientific information

## Abstract

Many urgent problems that societies currently face—from climate change to a global pandemic—require citizens to engage with scientific information as members of democratic societies as well as to solve problems in their personal lives. Most often, to solve their epistemic aims (aims directed at achieving knowledge and understanding) regarding such socio-scientific issues, individuals search for information online, where there exists a multitude of possibly relevant and highly interconnected sources of different perspectives, sometimes providing conflicting information. The paper provides a review of the literature aimed at identifying (a) constraints and affordances that scientific knowledge and the online information environment entail and (b) individuals' cognitive and motivational processes that have been found to hinder, or conversely, support practices of engagement (such as critical information evaluation or two-sided dialogue). Doing this, a conceptual framework for understanding and fostering what we call *online engagement with scientific information* is introduced, which is conceived as consisting of individual engagement (engaging on one's own in the search, selection, evaluation, and integration of information) and dialogic engagement (engaging in discourse with others to interpret, articulate and critically examine scientific information). In turn, this paper identifies individual and contextual conditions for individuals' goal-directed and effortful online engagement with scientific information.

## Introduction

Socio-scientific issues—from climate change to the ongoing COVID-19 pandemic (we will use the latter issue as an example in this article)—hold many consequences for personal, social, and civic life (Feinstein and Waddington, 2020). For such issues, defining the problem as well as coming up with possible solutions often rests on knowledge and evidence from the natural but also from the social sciences, which are well-beyond most citizens expertise (Zeidler, 2014). Nonetheless, most citizens want and need to stay informed and will likely seek information online, as searching for information on specific science-related issues is usually done on the Internet (National Science Board, 2018). In recent years, the percentage of people who use the Internet to learn about science has substantially increased, and there, they encounter a wide variety of digital media formats, including social media (Pavelle and Wilkinson, 2020). In this article, we review literature on the cognitive and motivational processes underlying *online engagement with scientific information* (OESI) that individuals employ in order to utilize the affordances and overcome the challenges of searching for and dealing with scientific information in online information environments.

**Figure 1:**
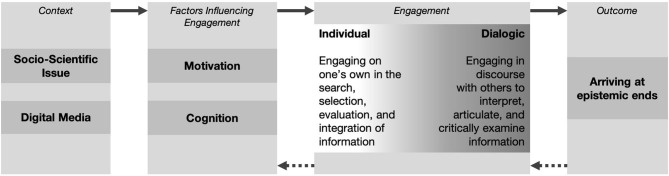
Heuristic model of online engagement with scientific information.

“Engagement” is an elusive concept but has been conceptualized as a behavioral manifestation of motivation or productive participation in a learning activity (e.g., Eccles and Wang, 2012; Bråten et al., 2018). Similar to previous models of engagement (Guthrie and Klauda, 2016), we understand OESI as *goal-directed* (that is, directed at achieving epistemic aims) and *effortful* activity in dealing with scientific information in online information environments, where this activity can be both *individual* and *dialogic*; is supported by *cognitive*, but also *motivational processes*; and leads to the individual arriving at *epistemic ends* (the target of epistemic aims). In the following, we describe our heuristic model in more detail (see Figure 1 for a graphical representation).

Central to our understanding of OESI is individuals' adoption of epistemic aims. In their AIR model of epistemic cognition, Chinn et al. (2014; see also, Chinn et al., 2011), identify epistemic *a*ims, *i*deals and *r*eliable processes that individuals apply to achieve epistemic ends. We describe all three components here briefly, before spelling out their relation to our notion of OESI. First, *epistemic aims* are “a subset of the goals people adopt, specifically those goals related to inquiry and finding things out” (Chinn et al., 2011; p. 142), and they are directed at achieving epistemic ends, for example, gathering “true” facts about a topic, avoiding misinformation on the topic, or acquiring a deeper understanding. Second, how much an epistemic end is valued will affect the selection of epistemic ends. An information seeker will review the success of an information search along her *epistemic ideals*, which could be described as the standard that determines whether a person has achieved her epistemic end; such a standard might be whether the information comes from a highly authoritative source or whether it is based on empirical evidence (Chinn et al., 2014; see also section Epistemic (meta-)cognition). And, third, to achieve epistemic ends, *reliable processes* are applied, which specify the conditions and cognitive operations to achieve reliable knowledge. Importantly, which processes are deemed reliable depends on the context and the individual's knowledge about the processes. For example, while observation is usually a reliable process to find things out about the (natural) world, individuals may overestimate the reliability of this process, which may lead to misconceptions (Chinn et al., 2014).

Epistemic aims underlie OESI and moderate transitions from stage to stage in our heuristic model (see Figure 1). First, when an individual is confronted with a socio-scientific topic in online media environments, which harbor specific constraints and affordances (see section Constraints and Affordances Entailed in the Context of OESI), this elicits cognitive and motivational processes, possibly leading the individual to form (an) epistemic aim(s). If so, these processes become more goal directed (as the individuals strives to arrive at an epistemic end). For example, if the individual adopts the epistemic aim of avoiding misinformation, she might consider more reliable processes in her search for information, such as referring to fact-check websites, which allow her to compare her achievements with her epistemic ideals (e.g., that accepted information must be evidence based). However, to adequately deal with context constraints and affordances (e.g., the amount of misinformation present in social media), the employed (reliable) processes must also be effortful. Such goal-directed and effortful engagement is what we describe as OESI, and we further differentiate individual engagement (engaging on one's own in the search, selection, evaluation, and integration of information) and dialogic engagement (engaging in discourse with others to interpret, articulate and critically examine scientific information).We assume that individuals will not follow a specific sequential order when engaging in these two types of engagement and their associated processes, but instead, depending on the situation and the individual's epistemic aim, any process could be the beginning of an episode of engagement and could lead to any other of the processes—within and between the two parts –, whereby the individual may even switch back and forth, commit to two processes at the same time, or skip a process. Finally, it is also possible for individuals to move back to previous stages: Practices of engagement may, in turn, motivate cognitive and motivational processes (e.g., if the individual feels self-efficient during critical information evaluation, she might be more motivated to achieve her epistemic aims). Furthermore, when the individuals arrives at her epistemic ends—or, instead, is partially or entirely unsuccessful in achieving her aim—she might reconsider her initial epistemic aims and enter another episode of engagement.

However, OESI may not lead to similar (and similarly measurable) achievements as does engagement in formal education settings. By defining outcomes as arriving at one's epistemic ends, we aim to highlight a central dilemma. Defining a successful outcome largely depends on which standards define achievement: personal (e.g., being content with a personal decision; relieving anxiety) or normative (e.g., achieving full understanding of a concept in alignment with the current scientific state of knowledge). We are aware that these aims require very different cognitive and motivational processes; consequently, we focus on engagement that is moderated by individuals' epistemic aims and we review research to find out which reliable processes are beneficial for achieving such aims, and for dealing with context constraints and affordances in the process [in contrast, Greene et al. (2020) recently focused on incidental learning in online environments]. Thus, the purpose of this article is to review the literature in several related fields in educational science and educational psychology to identify aspects of the context, and of individual's cognitive and motivational prerequisites that are especially beneficial or detrimental to effortful and productive OESI. Only when it is goal-directed and effortful can OESI lead to an individual successfully arriving at their respective epistemic ends.

Educational researchers and educational psychology researchers have long investigated individuals' reasoning and engagement with scientific and online information, and have posited educational implications; these researchers have delved much deeper into specific aspects relevant to our heuristic framework (e.g., Alexander and The Disciplined Reading and Learning Research Laboratory, 2012; Leu et al., 2013; Fischer et al., 2014; Tabak, 2015; Cho and Afflerbach, 2017; Breakstone et al., 2018; Britt et al., 2019; Coiro, 2020). Taking past conceptualizations into account, we use the term “online engagement with scientific information” not to introduce an entirely new concept or to replace any related concept; instead, here we review this literature, specifically to provide a comprehensive overview of OESI—focusing its context and on cognitive and motivational processes that support it—to derive implications for education and instruction.

### Constraints and Affordances Entailed in the Context of OESI

Information that we consider relevant for OESI is acquired in online environments and (a) contains an elaborate claim on a socio-scientific issue, or (b) is detailed enough to serve as evidence, or (c) both. For example, we would consider as relevant any text, audio, and video sources, as well as images and graphical representations (e.g., a tweet featuring a graph, a YouTube video, an open access scientific article), but we would not consider as relevant a meme consisting only of a photograph and some text, which is only meant to entertain. For individuals to deal with such information to achieve their epistemic aims, they must overcome the constraints and utilize the affordances that is entailed in the respective contexts (Barzilai and Chinn, 2019). We will briefly outline these in Table 1.

**Table 1:** Some context constraints and affordances of Online Engagement with Scientific Information (OESI).

	**Scientific knowledge**		**Online information environment**	
	**Constraints and affordances**	**Examples**	**Constraints and affordances**	**Examples**
Complexity	Complexity of knowledge in depth and breadth	Full understanding of the transmission of the SARS-Cov-19 virus requires knowledge from a variety of disciplines (e.g., infectology, virology, epidemiology) and relevant background knowledge from other disciplines (e.g., biology, chemistry). Above this, when deciding whether to re-open schools during a pandemic social science knowledge is required (e.g., from educational sciences)	Interconnected and embedded sources	A Wikipedia page includes hyperlinks to other Wikipedia pages. A science blog entry consisting of mainly text embeds pictures and graphs (embedded formats). A science-skeptic social media entry embeds a video of an interview with a scientist (hierarchical structure of formats and credibility cues).
Uncertainty	Uncertainty of evidence	A scientific measurement is imprecise. A scientific study cannot be replicated. It is yet unknown which long-term health effects remain after an infection with SARS-Cov-19. Scientists disagree about the effectiveness of a treatment.	Use of uncertainty to discredit science	Social Media entry advising against wearing cloth face masks, citing uncertainty about their effectiveness and uncertainty about adverse effects. An online newspaper article using balance reporting (devoting the same space to both sides of the issue) even though there is consensus within science.
Risks	Entailed risks on the personal and societal level	Health consequences of infection with SARS-Cov-19. Economic repercussions of the pandemic. Psychological effects of isolation during the pandemic. Educational effects of digital-only schooling.	Disinformation, misinformation and “fake news”	Disinformation: a member of the far-right deliberately posts on Facebook that scientists in China created the new Coronavirus. Misinformation: Someone shares this post considering it to be credible. “Fake news”: mimicking the layout of “real news” and sensationalizing (scientific) news to draw attention and promote sharing.
Level of Gatekeeping	High editorial gatekeeping, highly authoritative sources, limited access	Scientific journal articles authored by scientific experts (sometimes published as pre-print or open access). Reports by a selected group of experts (e.g., initiated institutions like the WHO or within scientific academies)	Low editorial gatekeeping, high diversity of sources, easy access	Science blog authored by a scientist. Journalistic article published on a newspaper's website. Youtube video by a person with a doctoral degree. Facebook entry by a layperson.
Communicative habits	Scientific genre	Almost all scientific publications are journal articles (often enhanced with representations). Most scientific journal articles follow a specific structure, style, format, and use of scientific jargon.	Variety of formats (e.g., text, video, representations), and genre (e.g., informational, narrative)	A scientist blogs about her study using comprehensible language. A Youtuber uses personalized language. A narrative video about virus reproduction.
Agency	Relevance to everyday- and societal questions	Immediate relevance of questions to behavior (e.g., washing hands, wearing a face mask), social and family life (e.g., visiting grandparents) and civic life (e.g., voting, protesting).	User agency	Users can decide what information to consider (e.g., by ordering an Email-newsletter or following certain social media accounts), but also how to consume it (e.g., free surfing, deciding to watch a video instead of reading a text).
Social Affordances	Argumentation as intrinsic to science	Social practices of science (e.g., conferences, peer-review, consensus building). Public Engagement with Science (Citizen Science, engaging members the public in generating research questions or funding decisions).	Social affordances, interactivity	Digital media entail affordances for immediate audience feedback and users' own active contributions: e.g., Like-button, comment section, discussion forum, creating own content.

Two characteristics of scientific knowledge are especially challenging for laypeople to deal with (Bromme and Goldman, 2014; Hendriks and Kienhues, 2019). First, scientific knowledge is characterized by complexity (Keil, 2008) as scientific theories vary in depth (deep causal complexity) and breadth (interrelatedness with other theories or concepts) (Bromme and Goldman, 2014). Consequently, full understanding of scientific phenomena requires both highly specialized knowledge in one field (e.g., virology) and related background knowledge from many other disciplines (e.g., biology, chemistry). For many questions in socio-scientific issues, the complexity of (natural) scientific knowledge is further amplified by manifold interrelations with the social sciences. This is especially the case when issues entail risk, which can exist both on a personal level (e.g., health risks) and on a societal level (e.g., economic risks). Second, scientific knowledge is intrinsically uncertain (Friedman et al., 1999), whereby uncertainty arises not only during evidence gathering processes (e.g., measurement error, inadequacies of measurement), but also from lack of knowledge or expert disagreement (van der Bles et al., 2019). Scientific uncertainty is becoming increasingly apparent to a larger public as the COVID-19 pandemic progresses, because evidence is rapidly accumulated and published online (sometimes before peer-review), such that public debates often involve highly uncertain scientific knowledge.

Both the complexity and uncertainty of scientific knowledge are amplified in online information environments. Online, there are many possibly relevant information sources that vary in format (e.g., text, video), in genre (e.g., scientific, journalistic, opinion, entertainment), and in explanatory power (e.g., relevant to the topic and founded in evidence). Moreover, sources are highly interconnected; that is, online documents not only embed and interlink diverse formats and genres (Alexander and The Disciplined Reading and Learning Research Laboratory, 2012; Goldman and Scardamalia, 2013), but interconnectedness is also established when sources cite and embed sources of different quality (e.g., when a scientist is interviewed by conspiracy-affiliated news sites), or when scientific arguments are disputed by industry stakeholders. To the individual, this amplifies the complexity of an already complex scientific topic. But also, scientific uncertainty can be amplified, especially as new and yet uncertain results are highly accessible online. In particular (digital), media pieces often display disagreement between experts (Boykoff and Boykoff, 2004), such as when scientists openly disagreed with statements by the WHO about the effectiveness of wearing face masks to protect against COVID-19 (Howard, 2020). Furthermore, around publicly contested issues like climate change and vaccination, skeptics have been especially strategic about utilizing uncertainty to manufacture doubt around scientific knowledge on the issue (Oreskes and Conway, 2011) and attack scientific evidence especially in digital media (e.g., Elgesem et al., 2015; Mercer, 2018).

As a result of these constraints, laypeople find it challenging to engage with scientific knowledge online to achieve epistemic aims; yet, the context of OESI also entails affordances that individuals can utilize. Socio-scientific issues may motivate individuals to purposefully engage with scientific information, because the scientific questions are highly relevant, and are often contextualized in everyday life and societal questions (Feinstein and Waddington, 2020). Science fundamentally rests on the active dialogue about and the critique of scientific claims (Osborne, 2010), and members of the public can now contribute more to this dialogue through efforts such as the movement toward Public Engagement with Science (Leshner, 2003). Furthermore, increased access to scientific information via digital media creates even more opportunities for individuals to connect with science (Brossard and Scheufele, 2013). Especially because scientific knowledge is often communicated in very formalized ways in terms of formats and language use, digital media platforms grant laypeople the opportunity to learn about science in various different formats and in much more accessible and engaging language; for example, YouTube videos often use an entertaining and narrative style to communicate quality informational content. However, because individuals can access such a wide variety of sources, they must be able to identify not only trustworthy sources, but also communicative intentions to distinguish, for example, institutional public relations information from critical science journalism, and even from science-related entertainment. Moreover, online, individuals must be especially aware of messages that are deliberately posted to disseminate false information, called *dis*information or “fake news” (a term that has also been weaponized in political contexts; Molina et al., 2019). In contrast, *mis*information is spread without malevolent intentions (Molina et al., 2019; Scheufele and Krause, 2019), but it is still a threat toward an individual's engagement with scientific claims and evidence.

The requirement to effortfully seek out credible information represent the downsides of individuals' ability to be active agents in using and interacting with online digital media platforms (Evans et al., 2016), where they can deliberately choose to engage with certain technologies, media, and content. Furthermore, individuals may even create their own content and—utilizing digital media's social affordances (Hopkins, 2016)—interact and engage in dialogue with other users.

In the article, we refer to research that describes which cognitive and motivational processes people employ to deal with these context constraints and affordances. While we do differentiate some constraints and affordances for the two contexts, some individuals may perceive an aspect that we introduced as constraint to be more of an affordance, and vice versa. For example, a comment section to a blog entry might initially be an affordance, but dealing with a high number of reader comments may hinder individuals' evaluation of information, thus making it a constraint.

## Individual Engagement

Searching for information to achieve epistemic aims is an iterative and dynamic process. To make sense of scientific information in order to achieve their epistemic aims on their own—to form “true” beliefs or understanding—individuals must employ reliable processes. To describe the necessary cognitive processes during an information search, we will first describe the MD-Trace (Multiple Documents-Task-based Relevance Assessment and Content Extraction) model (Rouet and Britt, 2011). According to this model, a search is initialized by an individual's mental representation of the searching task in a task model (see also, Rouet et al., 2017). Further, her task model also involves considering available knowledge and resources, such as prior topic knowledge and knowledge about search strategies (Rouet et al., 2017). As a result of these processes, the individual determines whether further information is needed to fulfill task demands and against what standard the search result should be compared. Having initiated the search process, she tests whether the sought information is relevant to her task model and *selects* documents accordingly. To *process* and evaluate the selected documents, the individual mentally represents them in an intertext model, which links contents of the documents to their meta-information (information about, e.g., the source, date, or rank of the search result), and includes intertext predicates (e.g., possible conflicts). *Integrating* information into the mental model allows the individual to coherently represent her acquired understanding of the issue. Finally, she may compare this integrated mental model against her initial task model to decide whether to redo certain steps of the search task or to go ahead with creating a search product (e.g., write an essay or make notes next to search results to further concretize a search task). However, at each step, individuals face several challenges (Rouet and Britt, 2011). In this section, we will summarize research on how searching, selecting, processing, and integrating scientific information are supported or hindered by aspects of the context and the individual's cognitive and motivational processes.

### Constraints and Affordances of the Online Information Environment to Individual Engagement

When searching for information, media affordances determine how specific technologies are used. That is, while users may deliberatively choose to use technologies or digital media for the potential features they offer; at the same time, such features also determine the ways in which users can engage with the technology. For example, when acquiring (scientific) information, individuals tend to use only one type of search engine, which might be enforced by the default use of digital assistants commonly installed on smartphones and computers (Kammerer et al., 2018). Additionally, characteristics of a search engine result page (SERP), such as the algorithm it uses to present search results, the interface it offers for users to manually filter search results, or the sparsity of information it displays (i.e., a title, short excerpt of the web page, and the URL) may influence whether an individual selects any of search results and whether they perform any further search queries. Research indicates that individuals would rather view the highest-ranked search results within a SERP (e.g., Salmerón et al., 2013; Haas and Unkel, 2017), even if those results are less relevant (Pan et al., 2007). Further, younger users in particular might select search results based on superficial cues like the search result's title (Lai and Farbrot, 2014), or boldface or capitalization (Rouet et al., 2011). Also, the number of documents that individuals select seems to vary by task: When individuals are asked to find a discrete answer to a question (instead of answering in an open-ended way), they select more documents (List et al., 2016a). Furthermore, individuals do not use all features of a search engine that perhaps would allow them to conduct more appropriate search inquiries. Kammerer and Gerjets (2014) found that interfaces displaying the results in a three-by-three grid more often led users to select and view search results according to their trustworthiness than according to their search rank. Similarly, Salmerón et al. (2010) found that individuals had more efficient reading times and displayed more explorative search behavior when using a graphical-overview interface (i.e., indicating the semantic relationships between the search results) instead of a standard list interface. Prior knowledge about the search topic may further benefit an individual during an information search when the search engine interface allows it: Experts performed faster and more accurate searches than laypersons when the interface was semantically structured (Salmerón et al., 2005).

Second, the interconnectivity and embeddedness of information sources—both hierarchically (documents that are interlinked), and horizontally (one document that is embedded within another)—may be challenging for information seekers to deal with (Cho and Afflerbach, 2017; Goldman and Scardamalia, 2013). These features call for flexibility in how individuals access information (Shapiro and Niederhauser, 2004), namely they have to access information in a non-sequential, non-linear way. This might require some specific aspect of digital literacy: Although expert searchers (fact checkers) were found to perform lateral reading, that is, opening several browser tabs during a search to check the reliability of a search result, this was not done by topic experts (historians) or students (Wineburg and McGrew, 2019).

The goal-directed and effortful evaluation of online information may further be constrained by several context features of scientific information in digital media environments (Breakstone et al., 2018; Forzani, 2019), such as genre, presentation of information (such as the use of distracting imagery), or other users' endorsements. Unfortunately, individuals often use only superficial or unreliable indicators for determining the credibility of online information (Coiro et al., 2015; McGrew et al., 2018). For example, individuals may not be able to distinguish sponsored news content from unbiased news stories or to identify the verified social media accounts of public organizations (McGrew et al., 2018). Furthermore, the extent to which adolescents use social media sites for entertainment purposes can be negatively related to their ability to discriminate reliable from unreliable online information (Macedo-Rouet et al., 2019b). Some online platforms, and especially social media, seem not to be regarded as trustworthy by individuals in general. Wikipedia is sometimes dismissed as information source without considering its inherent quality control (Breakstone et al., 2018). Evidence suggests that individuals deem Twitter and blog entries less trustworthy than (for example) newspaper articles and refrain from citing them, even if they entail relevant first-hand information about an issue (List et al., 2017).

Further, the communicative design of scientific information appears to affect its evaluation. Using a more “scientific” language style, such as including descriptions of scientific methods and in-text citations, leads readers to judge the information as more “scientific” and believable overall (Thomm and Bromme, 2012). Over a series of studies, Scharrer and colleagues (e.g., Scharrer et al., 2012, 2017) found that when a scientific text was written in a comprehensible fashion (compared to when the text contained technical terms and was, thus, incomprehensible for laypeople), readers were more easily persuaded by the text's arguments and less inclined to consult further expert advice. Furthermore, when individuals are engaged online in argumentation, presenting a piece of information in the form of question and answer rather than in the context of a traditional text may be a more effective way to promote the acquisition of factual knowledge (Iordanou et al., 2019a). The question-and-answer format appears to have facilitated learning, possibly by highlighting the potential use of a particular piece of information.

Another feature of online environments is that not only social media and blogs but also many online news sites allow for user comments, which might influence how users evaluate the content of the main article. For example, attitudes about a scientific issue may be influenced by the perceived consensus among other readers expressed through blog comments (Anderson et al., 2014; Lewandowsky et al., 2019). Furthermore, in some instances recommendations and social endorsements might play a role in evaluation and could reflect on evaluations of the credibility of health messages and of the expertise of the author (Jucks and Thon, 2017). In one study, when Facebook posts were shared by a close friend, this only raised the credibility of otherwise distrusted news sources (participants rated their trust in several news sources prior to reading the posts) but not of trusted sources (Oeldorf-Hirsch and DeVoss, 2020).

To sum up, during the first steps of searching for and selecting relevant information, characteristics of the online environment [e.g., (social) affordances of SERPs and digital media, communicative habits in digital media] may constrain, but also inspire effortful cognitive processes when searching, selecting and evaluating information. Dual-process theories propose that—unless task or person characteristics require it—individuals will default to heuristic processing instead of effortful and systematic processing (Salmerón et al., 2013). In an online information search, a variety of heuristic cues determine whether a search result is credible or relevant to the task at hand (Hilligoss and Rieh, 2008; Sundar, 2008; Metzger and Flanagin, 2013). Taraborelli (2008) stated that research has mainly focused on predictive judgments of credibility evaluation instead of evaluative judgments; this means that individuals may often engage in a first selection phase to sort out low-quality information in which superficial cues guide information selection, whereas in a second step they might engage in more effortful evaluation (Hilligoss and Rieh, 2008). In fact, in one study, individuals' first selection of search results relied on the order of appearance in a SERP, but they bookmarked more relevant pages to examine further (Salmerón et al., 2013). In another study, individuals did first select links by their titles, but on second glance they considered cues more indicative of information quality, like URLs and snippets with brief descriptions (Hautala et al., 2018).

However, the activity of online searching itself may lead to a feeling of knowing—the case when an individual perceives to possess knowledge but cannot actually retrieve it from memory (Pintrich, 2000; Koriat, 2012). Such an overestimation of acquired knowledge (Fisher et al., 2015) may result from representing the Internet as transactive memory (an external, collective memory system), leading one to better remember where a previously learned item is stored than to recall the item itself (Sparrow et al., 2011). Similarly, searchers might experience a “feeling of findability,” where they overestimate the availability of information online (Risko et al., 2016). These problematic assumptions may stem from a failure to distinguish “what is known” from “how was this knowledge acquired” (Kuhn, 1999). Such knowledge illusions may bias the integrated mental model of search results and thus, may negatively influence the integration of information into a coherent representation of the issue. As such, when misrepresenting acquired knowledge as a result of an online search, the individual might give up on an epistemic aim prematurely due to the assumption that it has been already resolved.

### Emotion and Motivation

Central to our understanding of OESI is identifying when individuals process information more effortfully instead of heuristically; importantly, the process of formulating epistemic aims and following through to resolve them might be strongly influenced by emotion and motivation. Referring back to dual-process theories, Griffin et al. (1999) identified several motivators for more systematic processing of information about risk. First, they found that the central motivators of information seeking were *information insufficiency*—when a person experiences a large gap between current knowledge and her personal sufficiency threshold (Griffin et al., 1999)—and a perceived normative pressure to be informed. Information insufficiency can follow affective responses to perceived risks (Dunwoody and Griffin, 2015). In fact, Yang and Kahlor (2013) found that while positive affect about climate change (e.g., hope) was related to information avoidance, negative affect (e.g., worry) was related to higher information insufficiency and the intention to seek information. Further, feeling personally threatened could bias how search terms are generated in an online search: Participants who were asked to reflect about a threat in their personal life generated more positive search terms in an unrelated Internet search than participants who were not instructed to think about personal problems (Greving and Sassenberg, 2015).

Similar notions end empirical evidence can be found in the literature on epistemic emotions, which are emotions directed at achieving epistemic ends (Muis et al., 2015). For example, enjoyment and curiosity may be positively related to the belief that justifying a knowledge claim requires critical evaluation, and anxiety and frustration may be lower when individuals believe that knowledge is uncertain (Muis et al., 2015). As such, different epistemic emotions may follow an experience of inconsistent or conflicting information. In fact, when individuals were surprised by incorrect answers in a trivia task (especially when their answers were given with high confidence) they had—as mediated by curiosity—more motivation to seek out explanations for these answers and request further information (Vogl et al., 2020).

In even more fundamental ways, the Cognitive Affective Engagement Model (CAEM) of multiple source use (List and Alexander, 2017b) addresses “learners' affective, cognitive, and behavioral involvement in multiple text use” (List and Alexander, 2017b, p. 184). Both situational and individual interest (Schiefele, 2009) have been found to promote learning and behavior (see also, Deci, 1992). Situational interest is a state that might be triggered by a single text (for example, when it is very easily comprehensible or coherent), while individual interest in a domain or topic is a trait-like personal characteristic (Schiefele, 2009). In consequence, the CAEM specifies an affective engagement dimension, which refers to an information seeker's interest and motivational involvement in the task at hand (also affected by topic-specific attitudes and prior beliefs), whereas the second dimension, behavioral dispositions, refers to the skills and strategies necessary for selecting, evaluating, and integrating information and documents at hand. By crossing these two dimensions, the CAEM states that learners fall into one of four default stances that guide their multiple-document comprehension: A “disengaged learner” selects and uses information without engaging much in evaluating and integrating. An “affectively engaged learner” accumulates information while engaging only in limited integration of multiple documents. An “evaluative learner” scrutinizes documents for relevance and credibility, but, due to limited motivational engagement, is less willing or able to fully integrate selected documents. A “critical analytic learner” possesses similar skills as the “evaluative learner” regarding source evaluation and verification, but since the critical analytical learner is highly motivated to engage in effortful and elaborate processing, they are able to succeed in integrating information into a coherent representation of the issue and, thus, might produce the most successful search result.

In sum, central motivators of goal-directed and effortful OESI are both personal relevance and topic specific risk perceptions (both affordances of socio-scientific topics). Furthermore, experienced information insufficiency may not be the only motivator to formulate epistemic aims; this may also be motivated by situational interest and epistemic emotions such as curiosity. Beyond individuals' skills to engage in reliable processes in dealing with scientific information, effortful evaluation and integration of information may also be fostered or constrained by emotions (both topic specific, e.g., hope or worry; and epistemic, i.e., directed at learning and understanding) and motivational involvement in the task.

### Epistemic (Meta-)Cognition

Epistemic beliefs have long been investigated as part of reasoning and arguing about scientific information. Such beliefs about the nature of knowledge and knowing (e.g., holding beliefs about scientific knowledge being uncertain, complex, or needing expert justification) may incite the use of reliable processes and strategies during OESI. Several studies in which students were asked to think aloud during an online search have demonstrated that students use their epistemic beliefs to define standards for learning and accordingly select their strategies (Hofer, 2004; Mason et al., 2010a,b, 2011; Barzilai and Zohar, 2012). For example, beliefs about the complexity of an issue led individuals to reflect on the need to compare several documents and collect contrasting views (Mason et al., 2011), and the belief that knowledge is given and stable did co-occur with less use of strategies to actively construct knowledge from texts (Bråten and Strømsø, 2006). A person's epistemological understanding ties in with her metacognitive processes and strategies (Kuhn, 1999; Muis, 2007; Barzilai and Zohar, 2016), as it may directly influence the standards she sets for acquiring knowledge and understanding (Muis, 2007). As such, Barzilai and Zohar (2016) have argued that epistemic metacognitive knowledge (as a specific part of metacognition) may “guide the execution of cognitive-level epistemic strategies as well as their selection, monitoring, and evaluation” (Barzilai and Zohar, 2016, p. 414).

Furthermore, epistemic beliefs may also affect how effortfully individuals execute practices of OESI. Evidence from studies using the think-aloud technique shows that epistemic beliefs influence individuals' abilities to engage in evaluating information both while navigating the web—e.g., identifying argumentative fallacies (Mason et al., 2010b)—and while reading (Ferguson et al., 2012; Iordanou et al., 2019b). Further, viewing knowledge as tentative enhances meaning-making as one deals with multiple documents (Bråten and Strømsø, 2010) and supports credibility assessment of newspaper articles, for example when they present simplified accounts of an issue (Strømsø et al., 2011). Individuals with evaluativist epistemic beliefs engage more often in evaluating the credibility of evidence presented in texts and use scientific research as their standard for judgment; for example, they might consider the number of scientific studies supporting a particular piece of evidence (Iordanou et al., 2019b). Besides supporting the evaluation of single pieces of information, adequate epistemic beliefs also support the evaluation and integration of multiple pieces of information presented in different sources (Bråten et al., 2011; Barzilai and Eshet-Alkalai, 2015). Empirical evidence shows that adequate epistemic beliefs support the integration of information during online learning (Barzilai and Zohar, 2012) and during reading of multiple texts (Ferguson and Bråten, 2013), where comprehension mediates the relationship between epistemic perspectives and information-source integration (Barzilai and Eshet-Alkalai, 2015).

In sum, beliefs about the nature of scientific knowledge may directly influence which strategies and practices are employed during OESI (Muis, 2007; Barzilai and Zohar, 2016), and may also affect the epistemic ideals by which epistemic ends are evaluated (Chinn et al., 2014). That is, in addition to an individual's scientific literacy (see section Evidence Evaluation and Scientific Literacy), her epistemic beliefs may inform how she assesses the uncertainty and complexity of scientific information, and these beliefs may also guide the selection and metacognitive regulation of reliable processes for achieving her epistemic aims.

### Source Evaluation

Due to limited gatekeeping of scientific information online (vs. editorial gatekeeping in scientific journals or traditional media), evaluating the source of scientific information is an especially important process within OESI, as it underlies the selection, evaluation, and integration of credible information. When retrospectively justifying document selection, students used epistemic criteria (e.g., source type, author) less often than non-epistemic criteria (e.g., order in the search list, relevance), but the more epistemic justifications were made, the more arguments and citations they presented in an open-ended search result (List et al., 2016b). However, individuals prefer authors of information to have good reputations (Rieh, 2002; Hilligoss and Rieh, 2008; Winter and Krämer, 2012); more specifically, readers tend to select blog posts by experts who possess relevant expertise on the topic in question (Winter and Krämer, 2014) and prefer disciplinary relatedness of search results to mere lexical similarity with search terms (Keil and Kominsky, 2013).

Diverse research findings suggest a variety of cues that individuals consider during source evaluation. First, the experts' language use seems to affect how trustworthy she is perceived. Individuals develop expectations about what constitutes appropriate language in different social and cultural contexts, and, thus, language accommodation or non-accommodation by speakers (reflecting their intentions and motives) may influence how individuals evaluate a speaker (Dragojevic et al., 2016). For example, an expert's use of technical language in scientific information may lead to her being ascribed higher expertise (Thon and Jucks, 2017) as well as higher integrity and benevolence when her use of (technical) language is appropriate to the context, e.g., when she uses less technical language when addressing laypeople (vs. experts) in online health forums (Zimmermann and Jucks, 2018), or less aggressive language in an online video (König and Jucks, 2019). Furthermore, the perception of a communicator in an online video as being comprehensible and entertaining also led to higher ascriptions of trustworthiness (Reif et al., 2020). Individuals also take an expert's motives into account when evaluating trustworthiness; for example, readers were more inclined to trust a scientist when they believed the scientist intended to inform rather than persuade them (Rabinovich et al., 2012), when the scientist provided a two-sided stance (instead of a one-sided stance) (Mayweg-Paus and Jucks, 2018) or mentioned the ethical aspects of a scientific issue (Hendriks et al., 2016). Furthermore, people perceived a source to be less trustworthy when the source had a vested interest in a claim (König and Jucks, 2019; Gierth and Bromme, 2020); this even sometimes motivated people to engage in effortful processing of complex evidence (Gierth and Bromme, 2020).

While these findings suggest that individuals are often able to adequately judge the trustworthiness of sources, research on “sourcing” (referring to when individuals pay attention to and use source features, such as the author, but also publication date) in multiple-document comprehension has found that students often fail to pay attention to source information (Britt and Aglinskas, 2002; Sandoval et al., 2016; for a review see, Brante and Strømsø, 2017). In fact, when evaluating multiple documents, individuals may not attend to author competence at all, and younger individuals (in elementary and middle-school) even failed to do so when explicitly prompted to evaluate sources (Macedo-Rouet et al., 2019a; Paul et al., 2019).

However, interacting with online information might not hinder successful sourcing *per se*. For example, reading an online document (instead of its printed-out version) increased memory for sources, which helped readers construct coherent interpretations of the issue at hand (Salmerón et al., 2017); that is, it helped them integrate information. Further, interacting with multiple sources is more effective than reading a single source for text comprehension and establishing source and content integration (e.g., Le Bigot and Rouet, 2007; Stadtler et al., 2013; Stang Lund et al., 2019); that is, individuals seem to have increased awareness about source information and create stronger content-source links when a conflict cannot be resolved by content information alone (Braasch et al., 2012; Strømsø et al., 2013; Stadtler and Bromme, 2014) or when information conflicts with prior beliefs about a topic (Bråten et al., 2016). As such, conflicts within single or multiple texts, as well as conflicts between newly acquired information and prior knowledge, might promote more effortful and strategic evaluation of sources (Braasch and Bråten, 2017). Further, relevant prior topic knowledge seems to benefit individuals' sourcing abilities (Stang Lund et al., 2019), whereas individuals with low prior knowledge may even prefer untrustworthy information sources (Bråten et al., 2011). In sum, while individuals use many different cues to determine source trustworthiness, encountering conflicting information about socio-scientific issues online seems to motivate individuals to engage in more effortful (source) evaluation and integration of information.

### Evidence Evaluation and Scientific Literacy

Evaluating the strength of evidence (or even its inner-scientific significance) should be central to individuals' consideration of information from a normative standpoint, but this is challenging for laypeople considering the uncertainty and complexity of scientific information and their own bounded understanding of science (Bromme and Goldman, 2014). One possibility to rate the credibility of scientific claims would be to assess argument strength and structure, for example whether a claim is backed by evidence. While laypeople adequately assess argument strength to be greater when it is supported by a greater amount of evidence (Corner and Hahn, 2009; Hendriks et al., 2020), they may sometimes not take prior studies into account when assessing the probability of an effect to be true (Thompson et al., 2020). Individuals might assume that the tentativeness included in scientific information means that the scientific results have limited credibility (Flemming et al., 2015); however, in one study that gave readers a refutation text alerting them that this assumption is wrong, the assumption was successfully reduced (Flemming et al., 2020). Similarly, a stronger epistemic belief regarding the uncertainty of science might alleviate the adverse effects of scientific tentativeness on the credibility of information (Rabinovich and Morton, 2012; Kimmerle et al., 2015). However, when making inferences from evidence, people may follow a causality bias, such as when interpreting correlational data (Shah et al., 2017). That is, new evidence may be rejected if it does not fit within a broader single causal framework (Koslowski et al., 2008). Further, it is unclear which type of evidence individuals consider to be informative. Although some studies have indicated that statistical evidence (citing a study), expert statements, and causal evidence are perceived to be more persuasive than anecdotal evidence (Hornikx, 2008), adding anecdotal stories into scientific news articles decreased the extent to which participants engaged in scientific reasoning about the evidence (Rodriguez et al., 2016). Moreover, individuals often do not take multivariate causality into consideration (Kuhn, 2020). Thus, successful online information behavior on complex topics is constrained by individuals' tendencies to think simplistically about complex issues instead understanding that most phenomena are caused by multiple contributing factors or, for judgments of a non-causal nature, taking multiple considerations into account (Kuhn and Iordanou, 2020).

Basic scientific literacy will also likely help individuals successfully evaluate and integrate scientific evidence. Internationally, educational frameworks for scientific literacy (e.g., OECD, 2017; National Research Council, 2012) have emphasized that a central aim of science education should be to familiarize students with processes of scientific inquiry, evidence evaluation, and argumentation. Scientific literacy has been ascribed three core dimensions: content knowledge (about a few core scientific concepts), procedural knowledge, and epistemic knowledge (Kind and Osborne, 2017). As such, it is important to consider how individuals understand not only the processes of doing science but also the modes by which it achieves reliable knowledge, such as expert epistemic practices (Golan Duncan et al., 2018). Kienhues et al. (2018) recently argued that “science-based arguments can be understood and judged by criteria on three layers of scientific knowledge: (1) the ontology, (2) the methods and sources, and (3) the social practices required for the generation and justification of the argument” (Kienhues et al., 2018, p. 253). They argue that everyday evaluation of scientific arguments may benefit from switching between these layers. For example, when it is not feasible to come to a conclusion about a scientific issue based on reliable evidence (maybe due to conflicting pieces of evidence), the individual may switch to investigating which scientific processes were used, which will help them identify which argument is backed by stronger evidence. If that is not feasible, the individual might judge whether the conflicting positions might be partly due to the complexity of the topic or the motivations of the involved experts behind the conflicting positions (Dieckmann et al., 2017; Thomm et al., 2017). Even if someone has limited content knowledge, they can still be successful in assessing a scientific issue online by determining, for example, whether there is consensus among scientists about an issue (a social practice of science; Oreskes, 2007; van der Linden et al., 2015) and then adopting the consensus view.

To summarize the two previous sections, individuals themselves often cannot adequately evaluate the credibility of a provided scientific claim, and some have argued that in such a case it is instead more feasible to evaluate the trustworthiness of the information source (Bromme and Goldman, 2014; Hendriks and Kienhues, 2019). That is, holding epistemic ideals regarding the justification of knowledge in consensus, or by a highly trustworthy source might be more beneficial for deciding whether to accept online information as provisionally true. Hence, instead of asking “What is true?,” individuals can rather solve the problem by asking “Whom do I believe?” (Bromme et al., 2010; Stadtler and Bromme, 2014). Hendriks et al. (2015) define epistemic trust as the willingness of a person to depend on an information source for knowledge; this trust is not blind, however, but relies on a person's epistemic vigilance toward cues that indicate whether an information source might be deceptive or ignorant (Sperber et al., 2010). In digital settings, evaluations of epistemic trustworthiness of expert sources rely on considering an expert's expertise (possessing relevant knowledge), integrity (adhering to the rules of their profession), and benevolence (having the interest of others at heart) (Hendriks et al., 2015).

### (Prior) Attitudes and Beliefs

Prior topic knowledge and attitudes can affect processes of individual engagement from the start of setting up a task model search to the (internal) formulation of a solution. On the one hand, prior topic knowledge and attitudes can result in individuals using more appropriate keywords and selecting more relevant information (e.g., MaKinster et al., 2002), on the other hand, they may also bias the information search. Selective exposure to information (sometimes referred to as *confirmation bias*) means that an individual is more likely to select attitude-consistent information (Fischer et al., 2005; Rothmund et al., 2015; Knobloch-Westerwick et al., 2020), and also evaluate that information more favorably (van Strien et al., 2016; Strømsø et al., 2017). An explanation for selective exposure during an information search might be defense goals, whereby an individual ignores or dismisses counter-attitudinal information to preserve their own worldview (Cappella et al., 2015; Winter et al., 2016). Nevertheless, those information seekers with high need for cognition are more likely to select two-sided information (e.g., suggested by the link title) for further reading (Winter and Krämer, 2012). Prior knowledge and attitudes may also detrimentally affect the evaluation and integration of scientific information online. Arguably, prior beliefs are internal representation with which newly acquired information has to be integrated. Richter (2015) assumes a “text-belief consistency effect” for integrating information into mental (situation) models. In fact, research shows that prior beliefs and attitudes might affect the way a person evaluates information and integrates new evidence into their internal representation of an issue. Chinn and Brewer (1998) showed that only in very few cases did anomalous evidence (evidence inconsistent with individuals' already established theories) result in careful consideration and adaptation of individuals' theories; often, such evidence was just ignored or discounted.

Motivated reasoning is also an important drive for rejecting information that is not consistent with the dominant belief in an individual's social group (Kahan, 2013). For example, group identity may cause individuals to apply defensive motivations when reading about scientific issues and, in consequence, might further strengthen the text-belief consistency effect (Maier et al., 2018). In one study, Nauroth et al. (2015) showed that people who self-identified with the social group of gamers devalued identity-threatening scientific information (e.g., playing video games increases violence in youth) that was presented in a science blog, and, when allowed to post a comment, they criticized the methodology of the scientific study. Further, in another study identity-threatening information affected how reputable and competent participants perceived the scientist authors to be (Nauroth et al., 2017). However, biased evaluation of scientific evidence may not only arise from an identity threat but also from a threat to one's general values. For example, the more central a person held non-violence to be in their self-concept, the more positively they evaluated a scientific study that claimed video games promote violence (Bender et al., 2016). Also, expert sources may be considered more credible when the ethical stance of the reader aligns with that of the source, leading to higher agreement with the source's claims (Scharrer et al., 2019).

In sum, prior beliefs and attitudes may play a central—and often detrimental—role in establishing a task model for searching for scientific information, as well as evaluating and integrating information. However, sometimes, prior beliefs may motivate effortful processing and evaluation of documents (Rouet and Britt, 2011; List and Alexander, 2017b; Rouet et al., 2017)—for example by eliciting curiosity by being unexpected (see section Emotion and Motivation) or evoking situational interest—allowing individuals to switch from belief protection to belief reflection (List and Alexander, 2017b). By judging the plausibility (“the potential truthfulness of a claim”; Sinatra and Lombardi, 2020, p. 5) individuals may utilize their prior knowledge by allowing them to select the most likely alternative, especially when an issue is contradictory and uncertain. Lombardi et al. (2016) provided a theoretical framework for plausibility judgments, which entail (a) alignment with prior knowledge and beliefs, (b) complexity of and (c) perceived conjecture within novel information, (d) judgments of source trustworthiness, and (e) the individual's heuristic processing and possible biases. Plausibility judgments may be guided by different degrees of evaluation. While most judgments are implicit (due to a preference for heuristic processing, see above), individuals' epistemic dispositions and motives (e.g., need for cognition) may lead to more effortful processing. Further, if motivated (e.g., if they are interested and self-efficient), individuals may also reappraise their original judgements, guided by more explicit processing and increased effort in reasoning. In consequence, Sinatra and Lombardi (2020) suggested that fostering individuals' capabilities to quickly make plausibility judgments about information—by efficiently employing their prior beliefs and knowledge—may be more fruitful in “post-truth” contexts (similar to the contexts we previously described for OESI) than training effortful strategies to evaluate information and its sources.

## Dialogic Engagement

Besides seeking and evaluating information independently to form beliefs, OESI includes engaging in discourse with others to share, interpret and critically examine scientific information. In this sense, social media platforms have emerged not only as an important source of information (Head and Eisenberg, 2010; Kim et al., 2014), but also as a public forum for engaging with science (Baram-Tsabari and Schejter, 2019). In fact, we perceive individual and dialogic engagement as reciprocal processes. For example, individually forming an understanding of an issue is immediately beneficial for constructing arguments when engaging in dialogue with others, and, conversely, dialogue and deliberation with others might one to revise their original understanding (see section Reciprocity of Dialogic and Individual Engagement).

When we consider OESI as a social process, it involves the overlapping processes of interpreting information, building arguments from that information and contrasting those arguments with competing arguments. Berland and Reiser (2009) propose that these processes, which they refer to as sensemaking, articulation and persuasion, respectively, form the foundation of scientific argumentation. Although scientific argumentation can be an individual process, as a dialogic process it presents a unique set of affordances and constraints. In the following sections, we explore these affordances and constraints and propose ways in which scientific argumentation as a social process can be leveraged to focus the epistemic aims and outcomes of OESI.

### Constraints and of Affordances of the Online Information Environment to Dialogic Engagement

Many different social media platforms exist, and their functions range from social networking and community building to collaborative knowledge construction and sharing (Leonardi, 2015; Krancher et al., 2018). Building on these potential functions, social media platforms may benefit the motivations and outcomes of OESI (Gao et al., 2012). However, to understand and to exploit the full potential of communication for using online information successfully with others on social media, we need to consider the role that a social media platform's characteristics play in users' abilities to select and establish network connections and to interact with other users (DeNardis, 2014). Following Ariel and Avidar (2015), the degree of interactivity is thereby not primarily determined by the technical features of a platform (interactivity as a medium characteristic) but rather by the actual aims and behaviors of its users (interactivity as a process-related variable). In other words, social networks such as Facebook, Twitter and Instagram do not necessarily produce interactive communication behavior *per se*, but rather they provide opportunities for different ways of communicative exchange.

In this regard, Rafaeli (1988) interactivity model suggests three possible types of messages in communication. The first type refers to one-way communication between a sender and a receiver, and messages are characterized by low responsiveness. The second type allows for two-way directional communication, as the receiver of a message becomes a sender and is, therefore, responsive to the information provided (or posted). However, only the third type enables real interactivity in a two-way flow of messages between users and is, therefore, highly responsive. Here, such interactive messages encourage the interaction to continue back and forth. Consequently, the transmission of information can be seen as the center of interaction, and interactivity seems to be a central attribute of the process of communication itself (Rafaeli and Ariel, 2007; Ariel and Avidar, 2015).

### Types and Goals of Dialogue

When we think about using information to communicate with others online, we should also think about the purpose of such communication. Two-way communication, or dialogue, can be divided into different types, each with a particular set of epistemic aims (Rapanta and Christodoulou, 2019). Walton (2010) identifies seven dialogue types that apply to communication in both face-to-face and online settings. These are information-seeking, discovery, inquiry, deliberation, negotiation, persuasion, and eristic dialogue (or “irrational dispute”). All are argumentative insofar as speakers posit how information can be brought to bear on claims, but they differ in their initial state and intended outcomes. For example, both inquiry and persuasion involve making claims with evidence, but inquiry focuses on collecting evidence to test claims, while persuasion focuses on citing claims and evidence to defend a conclusion. Dialogue types can also be distinguished by their social-emotional goals. To get at the role that personal stakes can play in dialogue, Asterhan (2013) proposes a distinction between *competitive interpersonal goals* and *collaborative interpersonal goals*. The former are competitive in that speakers take an adversarial stance on what they perceive to be zero-sum outcomes, and the latter are collaborative in that speakers take a cooperative stance on what they see as a shared enterprise. It is important to note that these interpersonal goal states are distinguishable from dialogue types. Some dialogue types may be more likely than others to trigger competitive interpersonal goals (persuasion, negotiation, and eristic, for example), while others may tend toward collaborative interpersonal goals (information-seeking, inquiry, and deliberation). However, interpersonal goals represent social-emotional outcomes that are distinct from the competitive or collaborative epistemic aims used to define dialogue types (except perhaps for eristic argument, which is primarily driven by interpersonal conflict). For example, negotiations can be conducted either collaboratively or competitively, depending on the stance, strategies, and dialogic moves chosen by each party (Lewicki et al., 2001). Likewise, although deliberations aim at group consensus, they may unfold as either collaborative or adversarial exchanges depending on the ways in which interpersonal dynamics emerge and are negotiated during dialogue (Tuler, 2000).

Based upon these considerations, we now focus on the potential benefits of argumentative dialogue as a two-way communication mode for addressing OESI in the context of dealing with (conflicting) scientific knowledge and socio-scientific issues. Numerous studies point out that dealing with complex content within argumentative dialogue has a positive effect on reasoning about information in online contexts [an overview is given in a meta-analysis by Noroozi et al. (2012)]. In order to successfully co-construct an elaborated understanding of an issue (e.g., Teasley, 1997; Chi, 2009), users need to apply “reasoning that operates on the reasoning of another” (transactivity, Berkowitz and Gibbs, 1983, p. 402). In this sense, transactive dialogue as a specific form a two-way argumentative requires coherent reference and mutual elaboration of each other's contributions by aiming at the integration of different knowledge backgrounds and perspectives (Asterhan and Schwarz, 2016). However, before well-elaborated consensus building is achieved, each contribution needs to be scrutinized critically (conflict-oriented consensus building; Fischer et al., 2002). Accordingly, an important feature of this type of consensus building is that individuals do not accept contributions of their partners as they are. In this context, efficient communication comprises strategies that directly address and challenge the argumentative structure and content (e.g., scientific evidence) of the other's contributions (Mayweg-Paus and Macagno, 2016; Mayweg-Paus et al., 2016a). In particular, critical questioning seems to be a strong argumentative strategy given its capacity to address deeper grounds of disagreement, bringing into light background knowledge and knowledge beliefs that might otherwise escape attention. In such cases, a goal is to avoid pseudo-agreements or pseudo-disagreements (Jucks and Paus, 2013) and to focus the discussion on the true source of differences in opinion. Consequently, asking critical questions seems to play a pivotal role in the context of knowledge construction (Chinn and Osborne, 2008) and for developing insights into not only science-related issues (Mayweg-Paus et al., 2016b; Thiebach et al., 2016) but also history (Wissinger and De La Paz, 2016) and public policy (Song and Ferretti, 2013).

When individuals hold one another accountable to standards for accurately collecting and interpreting information and validly using information as evidence, two-way communication offers distinct advantages over one-way communication. However, a two-way discussion can also undermine the quality of reasoning about evidence. The same set of forces that drive motivated reasoning when individuals think alone [see section (Prior) Attitudes and Beliefs] can also compromise reasoning when we engage in dialogue. Critical discussions, particularly those that polarize views on a topic (Kuhn and Lao, 1996), can prompt individuals to both overvalue confirming evidence and discount disconfirming evidence (Schulz-Hardt et al., 2002). This phenomenon is particularly concerning in Internet forums that attract users with polarized views on public issues [Baek et al., 2012; see also section (Prior) Attitudes and Beliefs]. Dialogue can also elicit adversarial behaviors that undermine the potential benefits of two-way communication. Thus, under some conditions, the competitive epistemic goals of persuasion can trigger competitive interpersonal goals that foreclose transactive dialogue (Asterhan, 2013; Felton et al., 2019). When speakers confuse the two goals, they tend to repeat themselves without elaborating their arguments, disagree without explaining why, and advance a barrage of arguments without addressing each other's counterarguments (Felton et al., 2015b). On the other hand, two-way communication can also trigger collaborative interpersonal goals that undermine dialogue. Several studies suggest that face threat can lead speakers to avoid critical discussion (See, e.g., Asterhan, 2013; Felton et al., 2019). The phenomenon may be particularly problematic when speakers encounter disagreement unexpectedly during in-group dialogue. In these circumstances, speakers are more likely to prioritize group or interpersonal cohesion over engaging in critical discussion and transactive dialogue (Concannon et al., 2015).

### Diverging Opinions and Dialogic Engagement

Collaboratively achieving epistemic aims in dialogic argument depends substantially on the discourse partners' efforts to deeply elaborate on and challenge their partner's knowledge and arguments (e.g., Kuhn and Udell, 2003). In this context, the dialogic character (or two-way mode) of argumentation can support OESI through (a) enhancing the quality of argumentation and the use of evidence (Crowell and Kuhn, 2014; Mayweg-Paus and Macagno, 2016) and (b) the evaluation and reconciliation of diverging claims (Nussbaum and Edwards, 2011; Felton et al., 2015a). In an argumentative dialogue, a person is subject to the interlocutor's scrutiny of her own position, which enhances her need to be more critical not only toward her own position but also the opposing one. In such dialogues, the reasons for preferring one point of view or one piece of evidence over another must be analyzed by taking a critical stance toward the presented evidence (Osborne et al., 2004). This challenge can only be addressed by drawing on more sufficient evidence and elaborating more and in greater depth on the different viewpoints and their backings (burden of proof, Walton and Macagno, 2007; Macagno and Walton, 2012).

There are several ways to address these potential challenges to effortful two-way, critical discussion. One effective strategy is to mitigate or de-emphasize competitive interpersonal goals by focusing attention on the epistemic aims of discourse. In the context of one-way communication, giving individuals specific instructions to generate reasons (Ferretti et al., 2000) or counter-arguments and rebuttals (Nussbaum and Kardash, 2005) have reduced my-side bias in writing when compared with instructions to persuade the audience. In two-way communication, focusing on collaborative epistemic aims (deliberation) as opposed to competitive epistemic aims (persuasion) in dialogue can lead to decreased interpersonal competitive behaviors and an increase in transactive dialogue (Felton et al., 2015b). These same conditions can also mitigate confirmation bias (Villarroel et al., 2016). However, it is important to note that in these studies, speakers were paired with someone who disagreed with them on the topic of discussion, and, therefore, the studies were designed to elicit the critical dialogue. But also, explicit expressions of disagreement in Youtube comment sections have the potential foster collaborative interaction (Dubovi and Tabak, 2020). What emerges in studies that compare competitive and collaborative epistemic aims is an optimization problem. Dissent is a valuable component in overcoming motivated reasoning, particularly when measures are taken to reduce the risk of losing face (Schulz-Hardt et al., 2006). Thus, dialogue can be structured to explicitly make room for dissent (Schulz-Hardt et al., 2006). However, cognitive engagement is an important ingredient in such conversations (Kuhn and Lao, 1996), and focusing on transactive dialogue aimed at epistemic aims enhances the quality of reasoning. Individuals must hold themselves accountable to expressing disagreement when it arises to avoid quick consensus while simultaneously focusing on collaborative interpersonal goals to promote transactive dialogue (Asterhan, 2013; Thiebach et al., 2016). Ultimately, collaboratively achieving epistemic aims involves focusing dialogue on epistemic aims while threading the needle of interpersonal goals to produce a social-emotional context that fosters critical discussion.

### Reciprocity of Dialogic and Individual Engagement

Collaboratively dealing with diverging (or even conflicting) claims might hold potential for the development of individual epistemological understanding, as it brings into light the existence of multiple perspectives and can promote a more balanced integration of pro and counter arguments in one's line of reasoning. Empirical evidence shows that individuals—after engaging in intervention studies that allowed them to engage in both argumentation with peers through the computer and in reflective activities for an extended period of time—showed improvements in their ability to evaluate others' arguments and the evidence that supported their arguments (Iordanou and Constantinou, 2015; Mayweg-Paus et al., 2016b). Further, engaging in an online discourse with peers who held an opposing view (vs. the same view) led to different inquiry behavior during online discussions and to different gains in terms of argument skills. In particular, in Iordanou and Kuhn (2020) study, individuals who engaged online in discussions with peers holding an opposing view chose to search for information regarding the opposing alternative first when given the opportunity. In contrast, individuals who engaged in online discussions with same-side individuals preferred to seek information related to their own position. Differences were also observed in the prevalence and types of functional evidence-based argumentive idea units in individual final essays, and they favored the students who engaged online in discussions with peers holding an opposing view. Here, engagement in online discussions with individuals holding opposing or same-side views may have fostered an epistemological understanding of recognizing that the other is reasoning from a perspective different from one's own, but that this perspective is still worth examining (Iordanou and Kuhn, 2020), or that it is important to take a step back and re-evaluate one's own understanding (Forzani, 2019). However, most people typically show difficulties with being able “to construct fully justified dual-position arguments and to explain and reconcile differences between accounts” (Barzilai and Ka'adan, 2017, p. 223). Apparently, recognizing multiperspectivity does not automatically mean one can apply sophisticated strategies when evaluating opposing views or arguments. Based on several empirical findings, Kuhn (2019) addresses this point by suggesting that understanding multivariable causality is a link toward evaluating and integrating multiple perspectives. Following this approach, OESI should include information- (or knowledge-) seeking activities for identifying and negotiating the multiple factors that can cause a phenomenon and to bring them into ongoing discussion.

In sum, dialogic engagement can take a number of forms depending on interactivity (one-way, two-way bounded, two-way unbounded), epistemic purposes (information seeking, discovery, inquiry, deliberation, negotiation, persuasion, eristic), and interpersonal dynamics of communication (collaborative, competitive). When we combine these variables, a complex array of permutations results. When individuals engage with others online about information, they gain access to critical dialogue that can enhance reasoning by focusing attention on the epistemic aims, ideals and reliable processes governing the use of information (Chinn et al., 2014). These epistemic concerns, when combined with critical dialogue, enhance reasoning about information and may even promote growth that transfers to individual engagement. However, to be successful in this endeavor, individuals must work collaboratively with others to examine their reasoning, even when epistemic aims are competitive.

## Conclusion and Implications

In this paper, we have addressed conditions that may benefit, but also hinder effortful online engagement with scientific information (OESI). The Internet offers users immediate access to a wide variety of information on socio-scientific issues, and also allows for user agency and interactivity. Coiro (2015) argued that, in theory, the Internet is an ideal place to engage with (scientific) information to achieve deeper learning and understanding and—from a reading perspective—she presents strategies learners need to achieve such epistemic aims. However, it is not feasible to assume that readers will allocate their cognitive and motivational resources to the systematic processing of all information they find online regarding an issue of interest (Stadtler, 2017). As such, our review collects literature on cognitive and motivational processes that may help individuals overcome constraints and utilize affordances of scientific information in the online environment. Before concluding this literature review with a discussion of our heuristic model of OESI, we elaborate on how to foster individual and dialogic OESI in (higher) education.

We have discussed several context factors that may both constrain and motivate effortful OESI. Dealing with the uncertainty and complexity of scientific knowledge (emphasized in online information environments) is a challenge that might be hard to overcome (Kienhues et al., 2020). In consequence, individuals might often encounter conflicts—of newly acquired information with their own beliefs, between information sources, or between their beliefs and those of their dialogue partner. As this review has shown, critical and deliberative scrutiny of information is central to OESI. That is, engagement should be directed at achieving epistemic aims while holding oneself and others accountable to appropriate epistemic ideals, at applying reliable processes in information search, selection, evaluation, integration, and in engaging in dialogue with others (in line with apt epistemic performance, Barzilai and Chinn, 2018). However, as we have discussed, cognitive biases (such as confirmation bias, motivated cognition, and competitive interpersonal goals) constrain otherwise reliable processes and may sometimes emerge under the guise of “critical thinking” (e.g., being critical toward experts' claims also has become a rhetorical device of science skeptics). As such, in order to counter one-sided reasoning and argumentation, open-minded thinking is directly beneficial to effortful OESI, because it entails the willingness to hold up all views (including one's own) under scrutiny of critical examination, even taking on the risk of identity threats, in order to follow through with epistemic aims (Taylor, 2016). Open-mindedness has been shown to not only benefit individual engagement with scientific information, such as knowledge about scientific issues and argument evaluation (Sinatra et al., 2003; Southerland and Sinatra, 2006; West et al., 2008), but also dialogic engagement in dialogue with others (Kuhn and Udell, 2003, 2007). We argue that it is through a balance of (individual or dialogic) critical examination of information and open-minded thinking that goal-directed and effortful OESI emerges. Sharon and Baram-Tsabari (2020) provide examples of several educational approaches to foster open-minded thinking, such as exposure to exemplars of virtues and practicing virtuous behaviors.

One environment that holds high potential for directly instructing critical and open-minded thinking by employing authentic search tasks is higher education classrooms. This environment is suitable for two reasons: First, students are already instructed to successfully deal with theories, models, evidence, and arguments within their discipline. Golan Duncan et al. (2018) identify that understanding experts' evidentiary practices (how experts analyze, evaluate, interpret, and integrate evidence to derive and inform theories) and being able to rely on scientific evidence even though one's own understanding of science is bounded (lay epistemic practices) are central for laypeople's ability to deal with scientific evidence. Searching for information on socio-scientific topics (related somewhat to a learner's area of expertise) is an ill-structured but solvable task, and it may also allow for reflection of the boundaries of students' expertise, especially when they are granted the opportunity to engage in dialogue with students from different disciplinary backgrounds or with diverging views on the issue. Second, while scientific inquiry tasks, such as lab work, are important to achieve procedural knowledge in their own discipline, there is limited opportunity for learners to engage in understanding of the social processes that are used to create reliable knowledge; however, both scientific knowledge as well as digital media entail social affordances allowing for dialogic engagement in authentic search tasks.

We have previously argued that the two parts—individual and dialogic engagement—are reciprocal rather than separate or sequential. While individual engagement might prepare the individual to engage in dialogue with others, such dialogic engagement might not only induce more individual engagement, but it may also foster skills and strategies needed for practices in individual engagement. Engaging learners in collaborative reasoning and argumentation about scientific information fosters individuals' epistemic cognition (e.g., Iordanou, 2016; Fisher et al., 2017), but it also creates a space to collaboratively reflect and elaborate on online scientific information in two ways: First, individuals may discuss the quality of online information, and, second, they may critically reflect and reason collaboratively on the criteria that guided their evaluations (Barzilai and Chinn, 2018). Thus, dialogue with others entails the potential to reflect on both one's own and others' individual engagement practices (Mayweg-Paus et al., 2020). In particular, to promote the development of epistemological understanding in their students, educational scholars need to address searching to learn as an information-seeking activity within the process of argumentation as well as learning to search in the context of argumentative dialogue (Redfors et al., 2014), which works as a mechanism for critical reflection on sourcing strategies, information providers, and media, and may also serve knowledge co-construction (Dubovi and Tabak, 2020). In this way, online dialogue becomes not only a medium for the transfer of information but also a means by which we gain epistemological insight into the nature of information and its many uses in our communication with others.

In our heuristic model, two aspects are not discussed in further depth. First, we decided not to define the cognitive and behavioral manifestations of the practices of engagement. Several descriptive models and literature reviews exist that describe one or several of these practices and their interrelations in more detail (in the context of multiple documents comprehension: e.g., Rouet and Britt, 2011; List and Alexander, 2017a; 2017b; epistemic cognition: e.g., Chinn et al., 2011, 2014; digital literacy: e.g., Cho and Afflerbach, 2017; Coiro, 2020; functional scientific literacy: e.g., Tabak, 2015). Second, we have not described how individuals would achieve their epistemic aims (the outcome of engagement), and whether it is always feasible to assume that individuals would always achieve these through goal-directed and effortful OESI. While there are models outlining knowledge integration with prior information (Richter, 2015), integration of diverging sources (Braasch and Bråten, 2017), and knowledge co-construction through collaborative dialogue and argumentation (Asterhan and Schwarz, 2016; Iordanou et al., 2019a), further research should investigate how knowledge construction takes place in authentic online information search (in contrast to dealing with provided information—often in text form—in a research or classroom setting), especially taking into account online-specific constraints and affordances. Newer studies have increasingly included combinations of process and outcome variables to more comprehensively examine online engagement (e.g., Bråten et al., 2014a,b; List and Alexander, 2018; Kammerer et al., 2020), or even tested theoretical models linking cognition, motivation, and learning (e.g., Muis et al., 2015). Furthermore, goal-directed and effortful OESI requires metacognitive knowledge and skills, such as current updates of the search task and monitoring of one's process (Barzilai and Chinn, 2018). A few studies have used think-aloud methods to investigate individuals' (epistemic) meta-cognition during online engagement (e.g., Mason et al., 2011; Barzilai and Zohar, 2012). While we think that such approaches should guide future empirical investigations into practices within OESI, our literature review also shows that there is ample research and evidence that future studies may build on.

Furthermore, our heuristic model of OESI could be extended in the future to include a larger variety of online information. Information technologies are constantly changing and with them users' access to information (e.g., on different devices, in different apps), information formats (e.g., interactive representations and video), information design (e.g., the use of nudges), and distribution (e.g., by algorithms, artificial intelligence). Hence, engagement with online information (and dealing with new and unique constraints and affordances) might already or will in the future involve even more steps, strategies, or skills (as well as many more variables mediating their effortful execution) than we have discussed in this review. Research on users' cognition and behavior in dealing with online scientific information—and especially on communication formats beyond informational text—is still sparse, but is growing in different disciplines (e.g., psychology, educational sciences, communication science, information sciences). We hope that future research would strive toward further integration of theoretical ideas and models within and across disciplinary bounds.

## Author Contributions

FH and EM-P conceived and presented the idea and structure of the article. FH took the lead in writing the manuscript. EM-P, MF, KI, and MZ each engaged in writing sections of the article. All authors provided feedback and ideas.

## Conflict of Interest

The authors declare that the research was conducted in the absence of any commercial or financial relationships that could be construed as a potential conflict of interest.
